# Proteomic profiling in cerebral amyloid angiopathy reveals an overlap with CADASIL highlighting accumulation of HTRA1 and its substrates

**DOI:** 10.1186/s40478-021-01303-6

**Published:** 2022-01-24

**Authors:** Andreas Zellner, Stephan A. Müller, Barbara Lindner, Nathalie Beaufort, Annemieke J. M. Rozemuller, Thomas Arzberger, Nils C. Gassen, Stefan F. Lichtenthaler, Bernhard Kuster, Christof Haffner, Martin Dichgans

**Affiliations:** 1 Institute for Stroke and Dementia Research (ISD), Klinikum der Universität München, Ludwig-Maximilians-Universität München, Feodor-Lynen-Straße 17, 81377 Munich, Germany; 2 Chair of Proteomics and Bioanalytics, Technical University of Munich (TUM), Freising, Germany; 3 Research Group Neurohomeostasis, Department of Psychiatry and Psychotherapy, University of Bonn, Bonn, Germany; 4 German Center for Neurodegenerative Diseases (DZNE), Munich, Germany; 5 Neuroproteomics, School of Medicine, Klinikum rechts der Isar, Technische Universität München, Munich, Germany; 6 Department of Pathology, Amsterdam Neuroscience, VU University Medical Center, Amsterdam, The Netherlands; 7 Department of Psychiatry and Psychotherapy, Klinikum der Universität München, Ludwig-Maximilians-Universität München, Munich, Germany; 8 Munich Cluster for Systems Neurology (SyNergy), Munich, Germany; 9 Present Address: Department of Psychiatry and Psychotherapy, School of Medicine, Klinikum rechts der Isar, Technische Universität München, Ismaninger Str. 22, 81675 Munich, Germany

**Keywords:** Cerebral amyloid angiopathy, Cerebral small vessel disease, CADASIL, Proteomics, HTRA1, Proteostasis

## Abstract

Cerebral amyloid angiopathy (CAA) is an age-related condition and a major cause of intracerebral hemorrhage and cognitive decline that shows close links with Alzheimer's disease (AD). CAA is characterized by the aggregation of amyloid-β (Aβ) peptides and formation of Aβ deposits in the brain vasculature resulting in a disruption of the angioarchitecture. Capillaries are a critical site of Aβ pathology in CAA type 1 and become dysfunctional during disease progression. Here, applying an advanced protocol for the isolation of parenchymal microvessels from post-mortem brain tissue combined with liquid chromatography tandem mass spectrometry (LC–MS/MS), we determined the proteomes of CAA type 1 cases (n = 12) including a patient with hereditary cerebral hemorrhage with amyloidosis-Dutch type (HCHWA-D), and of AD cases without microvascular amyloid pathology (n = 13) in comparison to neurologically healthy controls (n = 12). ELISA measurements revealed microvascular Aβ_1-40_ levels to be exclusively enriched in CAA samples (mean: > 3000-fold compared to controls). The proteomic profile of CAA type 1 was characterized by massive enrichment of multiple predominantly secreted proteins and showed significant overlap with the recently reported brain microvascular proteome of patients with cerebral autosomal-dominant arteriopathy with subcortical infarcts and leukoencephalopathy (CADASIL), a hereditary cerebral small vessel disease (SVD) characterized by the aggregation of the Notch3 extracellular domain. We found this overlap to be largely attributable to the accumulation of high-temperature requirement protein A1 (HTRA1), a serine protease with an established role in the brain vasculature, and several of its substrates. Notably, this signature was not present in AD cases. We further show that HTRA1 co-localizes with Aβ deposits in brain capillaries from CAA type 1 patients indicating a pathologic recruitment process. Together, these findings suggest a central role of HTRA1-dependent protein homeostasis in the CAA microvasculature and a molecular connection between multiple types of brain microvascular disease.

**Supplementary Information:**

The online version contains supplementary material available at 10.1186/s40478-021-01303-6.

## Introduction

Cerebrovascular pathologies are a major cause of stroke, cognitive decline, and dementia thus posing a significant burden for aging societies [17, 27, 59]. Disorders of the brain microvasculature, collectively termed cerebral small vessel diseases (SVDs), are particularly common in the aging brain and encompass a variety of sporadic and hereditary conditions affecting small and medium-sized vessels in the cerebral cortex, subcortical white matter, and deep white matter [48, 68]. Among the most frequent pathologies is cerebral amyloid angiopathy (CAA), an important cause of intracerebral hemorrhage and cognitive decline [2, 13]. Vascular amyloid is also seen in a substantial proportion of patients with Alzheimer’s disease (AD) [20]. CAA is characterized by the misfolding and excessive vascular deposition of amyloid-β (Aβ) peptides, which are generated by multiple proteolytical processing of the β-amyloid precursor protein (APP) [60]. While mostly occurring as a sporadic condition, CAA can also develop from rare *APP* mutations such as the E693Q mutation, which causes hereditary cerebral hemorrhage with amyloidosis-Dutch type (HCHWA-D) [33].

Cerebrovascular amyloid deposits can be observed in both large (e.g. leptomeningeal) vessels and small parenchymal arterioles and capillaries, with their presence in capillaries determining the classification of CAA into two subtypes. Type 1 is defined by the presence of Aβ pathology in capillaries and may show additional Aβ deposition in non-capillary blood vessels. In contrast, Aβ pathology in type 2 is restricted to leptomeningeal and cortical arteries and arterioles [58]. Vascular Aβ accumulation is primarily considered a consequence of reduced clearance of parenchymal Aβ, for which different mechanisms are discussed depending on vessel type: while an impairment of transendothelial clearance is regarded as the predominant pathological process in capillaries, a reduction in perivascular Aβ clearance might prevail in larger blood vessels [2, 69]. The capacity of these physiological clearance pathways is believed to decline with age and under pathological conditions resulting in incomplete Aβ elimination and its focal build-up at different locations within the vasculature [12, 50]. Capillary CAA is frequent in AD [3], is associated with both microvascular occlusions and disturbances of cerebral blood flow [57], and might contribute to cognitive decline in AD [24]. An invariant histopathological feature in CAA patients is a disruption of the vascular architecture including mural cells loss [21, 32, 65, 66] and alterations of the extracellular matrix (ECM) with thickening, splitting and duplication of the basement membrane [43].

The molecular mechanisms linking vascular Aβ deposition to vessel pathology and dysfunction remain largely elusive. However, recent proteomic studies have identified a variety of proteins and pathways with a possible role in CAA pathophysiology. Two studies isolated leptomeningeal and large cortical vessels from cryopreserved post-mortem tissue by manual dissection or laser-capture microdissection [28, 38]. A third study used biopsy material from patients undergoing surgery for large lobar hemorrhages [18]. Collectively, these investigators found several proteins including apolipoprotein E (APOE), clusterin (CLU, also known as apolipoprotein J) and vitronectin (VTN) to be enriched in vessels from CAA patients and to co-localize with Aβ deposits evidencing co-aggregation or recruitment of these proteins into Aβ deposits. APOE and CLU were further shown to interact with Aβ and to influence its aggregation and clearance in an in vitro model of CAA that recapitulates the intramural periarterial drainage process [18]. In a fourth proteomic study, microdissected cortical tissue showing either parenchymal or vascular Aβ pathology from AD patients with or without capillary CAA was used and norrin and collagen VIα2 (COL6A2) identified as highly selective CAA markers [25].

Despite the abundancy and presumed functional relevance of vascular amyloid pathology in the brain [45], the specific molecular changes in small parenchymal vessels from CAA type 1 patients remain insufficiently characterized. Recent advances in the protocols for isolating brain microvessels from autopsy samples have facilitated their targeted biochemical analysis [6, 42, 72]. For instance, Bourassa and colleagues examined microvasculature-enriched fractions from human cerebral cortex by a combination of Western blot, enzyme-linked immunosorbent assay (ELISA), and immunofluorescence analyses and found CAA patients to exhibit alterations in the abundance of several endothelial markers and of proteins involved in Aβ production and clearance [6]. To our knowledge, however, there have been no focused efforts to characterize the full range of proteomic changes in parenchymal microvessels from CAA type 1 patients.

Applying untargeted proteomics to isolated brain microvessels, we recently assessed the microvascular proteome of CADASIL, a monogenic form of ischemic SVD that is caused by mutations in the NOTCH3 gene and characterized by vascular deposition of the Notch3 extracellular domain (Notch3^ECD^) [72]. This study demonstrated a distinct proteomic profile characterized by the accumulation of multiple proteins. Among the most strongly enriched proteins was the serine protease high-temperature requirement protein A1 (HTRA1). Moreover, many of the accumulating proteins were found to be HTRA1 substrates suggesting a loss-of-function signature. Notably, loss-of-function mutations in HTRA1 cause yet another form of hereditary SVD [23, 63]. We further demonstrated co-localization of HTRA1 with Notch3^ECD^ deposits and an accumulation of various HTRA1 substrates in brain parenchymal microvessels from CADASIL patients. Collectively, these findings provided evidence for a loss of HTRA1 proteolytical function as a critical step in CADASIL pathogenesis.

The current study aimed to characterize the proteomic profile of brain parenchymal microvessels in CAA type 1 to identify key molecular targets implicated in disease pathogenesis. The analysis resulted in the detection of a distinct CAA type 1 profile encompassing multiple secreted proteins and ECM constituents. This profile, which was not observed in AD cases, showed a remarkable overlap with the brain microvascular proteome of CADASIL [72] highlighting the accumulation of HTRA1 and several of its substrates. We further identify serum amyloid P component (APCS) and PRSS23, another serine protease, as novel HTRA1 substrates. Our findings suggest a role of HTRA1 in CAA type 1 pathogenesis thus further highlighting shared mechanisms among distinct types of cerebral SVD.

## Materials and methods

### Human brain tissue

Cryoconserved human brain autopsy samples (cortex and adjacent white matter from occipital or parietal lobe) from 12 neuropathologically confirmed CAA patients, 12 neurologically healthy control subjects and 13 neuropathologically confirmed AD patients were obtained from the Netherlands Brain Bank (Netherlands Institute for Neuroscience, Amsterdam). All CAA cases had been classified as capillary type (type 1) by routine neuropathological autopsy examination.

### Microvessel isolation

Microvessels were isolated from 100 mg of frozen brain tissue as previously described [72]. In brief, brain tissue was minced and homogenized in cold minimum essential medium using a glass tissue grinder (Wheaton). After adding Ficoll to a final concentration of 15% and centrifugation at 6000 × g for 20 min at 4 °C, the resulting pellet was resuspended in 1% bovine serum albumin (BSA) diluted in PBS, transferred onto a nylon mesh (40 µm), and extensively washed with cold PBS. Microvessels were collected by flushing the inverted nylon mesh and centrifugation at 3000 × g for 5 min. Purity was checked by light microscopy. Throughout the procedure, plastic material was coated with 1% BSA in PBS.

### Immunofluorescence staining

Immunofluorescence staining of isolated vessels was performed as previously described [72]. In brief, microvessels were transferred onto a microscope slide and air-dried at room temperature (RT). After fixation and permeabilization with 100% acetone for 10 min at − 20 °C, slides were washed with PBS, blocked with 5% BSA in PBS for 1 h at RT and then incubated with primary antibody (diluted in 0.2% BSA in PBS) overnight at 4 °C. The same protocol was used for the staining of tissue cryosections (16 µm). The following primary antibodies were used: Goat polyclonal anti-collagen IV (#1340–01, SouthernBiotech, 1:1000), mouse monoclonal anti-HTRA1 (MAB2916, R&D, 1:50), rabbit polyclonal anti-Aβ (clone 3552, 1:200, kind gift from H. Steiner, C. Haass). Subsequently, slides were washed with PBS and probed with the respective fluorophore-conjugated secondary antibody (Alexa Fluor 488-, Cy3- or Alexa Fluor 647, Abcam 1:500) for 1 h at room temperature. After washing with PBS, vessels or sections were mounted (Fluoromount, Sigma-Aldrich) and images were captured by confocal microscopy (LSM800, Zeiss).

### Protein extraction and Aβ ELISA

Protein extraction of isolated microvessels was performed in a buffer containing 4% SDS, 100 mM Tris–HCl pH 7.6, 100 mM DTT. Samples were processed by Precellys tissue homogenizer (5 × 30 s, 10,000 rpm, 30 s pause), heated for 3 min at 95 °C and subsequently sonicated (5 × 30 s, amplitude 100%, duty cycle 50%) with intermediate cooling using the VialTweeter sonicator (Hielscher). Lysates were cleared by centrifugation at 16,000 × g for 15 min, supernatants collected, and protein concentration determined using the colorimetric 660-nm assay according to the manufacturer’s instructions (Thermo Fisher Scientific). Aβ_1-40_ and Aβ_1-42_ species were quantified using the V-PLEX Plus Aβ Peptide Panel 1 (6E10) enzyme-linked immunosorbent assay (ELISA) Kit (Meso Scale Diagnostics) according to the manufacturer’s instructions. Samples were diluted 1:100 and measured in triplicates. The resulting Aβ levels were normalized to total protein concentrations.

### LC–MS/MS

A protein amount of 30 µg vessel lysate was subjected to proteolytic digestion by a modified single-pot solid-phase-enhanced sample preparation (SP3) protocol [26]. Resulting peptides were desalted, dried by vacuum centrifugation and dissolved in 20 µL 0.1% formic acid. A micro-flow LC–MS/MS system composed of a modified Dionex UltiMate 3000 RSLCnano System coupled to a Q Exactive HF-X mass spectrometer (Thermo Fisher Scientific) was used for all proteomic measurements in this study, as described in detail by Bian et al. [5]. Peptides (4.0 µg) were separated on a 15 mm long C18 column with an inner diameter (ID) of 1 mm (Acclaim PepMap RSLC, Thermo Fisher Scientific). A binary 60-min gradient of water (A) and acetonitrile (B) containing 0.1% (v/v) formic acid and 3% DMSO was applied as follows using a flow rate of 50 µL/min: 0 min, 0.5% B; 0.2 min, 2% B; 52.7 min, 24% B; 60.2 min, 35% B; 60.5 min, 90% B; 62.7 min, 0.5% B. Sample loading and column wash was performed at an increased flow rate of 100 µL/min. The column was heated to 55 °C. MS1 spectra were acquired at a resolution of 120,000, a scan range from 360 to 1400 m/z, a maximum injection time of 100 ms and an AGC target of 3E6. The Top 20 precursors were subjected to higher-energy c-trap dissociation with a normalized collision energy of 28%. A resolution of 15,000, an AGC target of 1E5, an isolation window of 1.6 m/z and a maximum injection time of 22 ms was applied. The dynamic exclusion was set to 30 s.

### Proteomic data analysis

The data were analyzed with the Maxquant software [16] version 1.6.17.0 and searched against a reviewed canonical FASTA database of Homo sapiens (UniProt, download: June 26th 2021, 20,395 entries). To recalibrate the peptide masses within a window of 20 ppm, the option first search was used. Main search was performed for peptides and peptide fragments within a mass tolerance of 4.5 and 20 ppm respectively. N-terminal acetylation and oxidation of methionine were set as variable, carbamidomethylation of cysteine as static modification. The false discovery rate (FDR) was adjusted to less than 1% for both peptides and proteins. For label-free quantification (LFQ) of proteins, at least two ratio counts of unique peptides were required. To determine the significance of protein abundance changes between the different groups, LFQ intensities were log_2_-transformed and a two-sided Student’s *t*-test was applied. Relative quantification and statistical analysis were performed for proteins identified in at least six samples of each group using the following significance threshold: *p* value < 0.05 and log_2_ LFQ ratios > 1.0 and < − 1.0 (corresponding to 2.0-fold and 0.5-fold changes). The R software packages GOChord and SuperExactTest (version 1.0.4) [67] were used for generating the circos diagram and for statistically assessing the proteomic profile overlaps.

### HTRA1 proteolysis assays

To investigate HTRA1-mediated proteolysis of APCS and PRSS23, human embryonic kidney cells (Expi293, Thermo Fisher Scientific) maintained in Expi293 Expression Medium (Gibco, Thermo Fisher Scientific) at 37 °C, 8% CO_2_ and 125 rpm were transiently transfected with pTT3/APCS-Bio/His (gift from Gavin Wright [55], Addgene #53424), pcDNA4/TO/PRSS23-Myc/His (SourceBioscience) and pcDNA6/HTRA1-V5/His expression plasmids using ExpiFectamine 293 (Thermo Fisher Scientific). 48 h after transfection, conditioned supernatants were collected by centrifugation at 1000 × g for 5 min and co-incubated at a substrate to protease ratio of 5:1 (v/v) for 24 h at 37 °C. Afterwards, protein intensities were analyzed by western blotting. The HTRA1-specifc inhibitor NVP-LBG976 (Novartis) [19] was used for the co-incubation at a final concentration of 5 µM.

### Western blot analyses

Conditioned cell culture supernatants were analyzed by sodium dodecyl sulfate–polyacrylamide gel electrophoresis and electrotransfer onto 0.2-µm nitrocellulose membranes using the Mini-Protean and Trans-Blot system (Biorad). Membranes were blocked with 4% skim milk powder dissolved in Tris-buffered saline supplemented with 0.1% Tween (TBS-T) for 1 h at RT and then incubated with sheep polyclonal anti-APCS (AF2558, R&D 1:2000), mouse monoclonal anti-Myc (9E10, Santa Cruz Biotechnology, 1:4000) or mouse monoclonal anti-V5 (#R960-25, Thermo Fisher Scientific, 1:10,000) primary antibody (diluted in blocking buffer) overnight at 4 °C. Subsequently, blots were washed and probed with horseradish peroxidase-conjugated anti-mouse (Cell Signaling) or anti-sheep (R&D) secondary antibody diluted 1:10,000 in blocking buffer for 1 h at RT. Immuno-reactive bands were visualized using chemiluminescence development (Immobilon ECL detection reagent, Merck Millipore) and the Fusion FX7 imaging system (Vilber Lourmat).

## Results

### Sample characterization

**Fig. 1:**
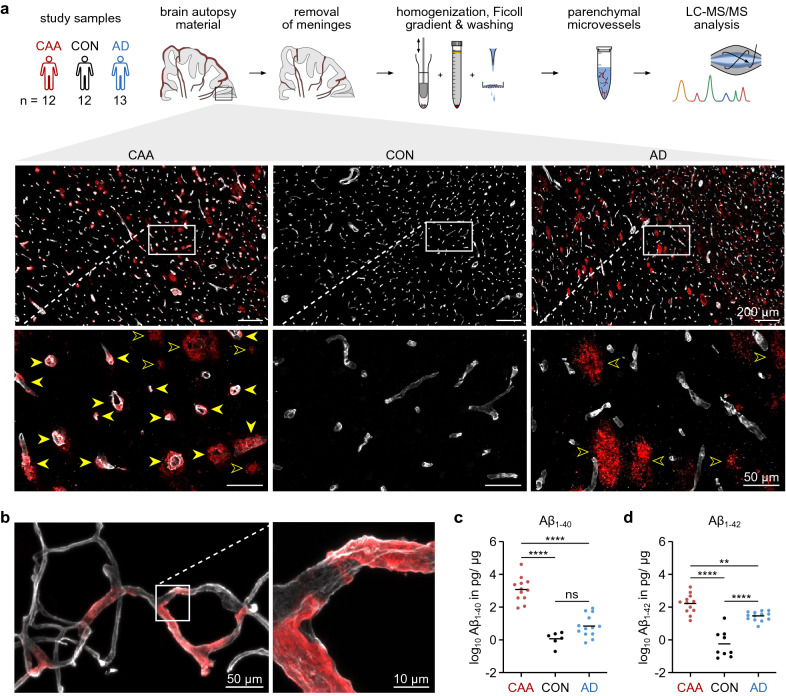
Study workflow and characterization of microvessel preparations used for the proteomic analysis. **a** Study workflow (top): Parenchymal microvessels were isolated from cryopreserved post-mortem brain samples of patients with cerebral amyloid angiopathy type 1 (CAA), control subjects (CON) or patients with Alzheimer’s disease (AD) and analyzed by LC–MS/MS. Confocal microscopy images (bottom) of a brain tissue section of a representative CAA, control and AD case immunostained for the amyloid beta (Aβ) peptide (red) and for the basement membrane marker collagen IV (pseudocoloured in white). Prominent vascular Aβ immunoreactivity in arterioles and capillaries (indicated by filled yellow arrows) are only observed in CAA. Parenchymal amyloid plaques, indicated by empty yellow arrows, are observed in CAA and AD. **b** Confocal microscopy image of a capillary network isolated from a CAA patient with hereditary cerebral hemorrhage with amyloidosis-Dutch type (HCHWA-D) and stained for Aβ (red) and for the basement membrane marker collagen IV (pseudocoloured in white). **c**, **d** Level of the amyloid species Aβ_1-40_ (**c**) and Aβ_1-42_ (**d**) in the microvessel extracts determined by ELISA. *p*-values were calculated using ANOVA with Tukey post hoc analysis, **p = 0.0078, ****p < 0.0001. Of note, the HCHWA-D case showed the highest levels of both Aβ_1-40_ (40,929 pg/μg) and Aβ_1-42_ (1699 pg/μg) across all samples

Cryopreserved post-mortem brain samples from patients with cerebral amyloid angiopathy (CAA) type 1 (n = 12, mean age 80.5 ± 9.8 years), neurologically healthy controls (n = 12, mean age 79.8 ± 8.0 years) and patients diagnosed with Alzheimer’s disease (AD) (n = 13, mean age 77.6 ± 8.5 years) (Table 1) were obtained from the Netherlands Brain Bank. To verify the presence or absence of Aβ pathology we performed immunofluorescence staining of tissue sections of selected cases along with confocal microscopy imaging (Fig. 1a). CAA samples showed extensive co-localization of Aβ immunoreactivity with parenchymal arterioles and capillaries demonstrating a high microvascular Aβ load whereas there was no microvascular Aβ immunoreactivity in the AD cases, which in turn were characterized by parenchymal plaque staining. Control samples were devoid of any Aβ immunoreactivity. Next, we validated our recently established brain microvessel isolation protocol [72] (see workflow in Fig. 1a) in one of the CAA cases. Confocal microscopy imaging of vessel preparations stained for the basement membrane marker collagen IV and for Aβ revealed the presence of intact capillaries and enabled visualization of vascular amyloid deposits in high resolution (Fig. 1b). Aβ immunoreactivity was mostly detectable as patches of variable size in confined vessel segments. To obtain a quantitative measure of the load of Aβ_1-40_ and Aβ_1-42_ peptides in our samples, we performed ELISA measurements on the same microvessel lysates prepared for liquid chromatography/tandem mass spectrometry (LC–MS/MS) analysis. Aβ_1-40_ and Aβ_1-42_ were massively enriched in CAA samples (mean: 3226-fold for Aβ_1-40_ and 111-fold for Aβ_1-42_ compared to controls) and showed the highest level in the HCHWA-D case (Fig. 1c, d and Additional file 1: Table 1). In contrast, AD cases exhibited isolated enrichment of Aβ_1-42_ (mean: 11-fold compared to controls).

**Table 1:** Autopsy sample characteristics

	Age	Sex	Brain region	PMD	CAA (Thal)	Aβ (Braak)	Tau (Braak)	CERAD	APOE
CAA 1	88	f	par	05:00	2	A	2	NA	42
CAA 2	81	m	par	06:30	2	C	5	2	44
CAA 3	71	m	occ	04:00	2	C	6	3	43
CAA 4	78	f	par	04:20	3	C	3	0	44
CAA 5	89	f	occ	07:35	2	C	5	2	43
CAA 6	70	f	par	04:00	3	C	6	3	33
CAA 7	80	f	par	05:10	2	C	4	2	43
CAA 8^$^	71	m	par	07:15	2	A	1	0	32
CAA 9	97	m	occ	05:10	3	C	5	3	43
CAA 10	65	m	occ	04:30	2	O	0	0	33
CAA 11	88	m	par	03:55	3	C	5	3	44
CAA 12	88	m	occ	05:00	2	C	4	2	43
CON 1	84	m	par	05:35	0	A	1	0	33
CON 2	84	f	par	04:45	0	O	1	0	33
CON 3	80	m	par	06:30	0	A	0	0	33
CON 4	85	m	par	04:15	0	O	1	0	44
CON 5	72	f	par	06:50	0	A	1	0	33
CON 6	88	f	par	05:55	0	A	1	0	33
CON 7	75	f	occ	09:10	0	A	1	0	32
CON 8	61	f	occ	06:50	0	O	0	0	32
CON 9	82	f	occ	07:00	0	O	1	0	33
CON 10	84	m	occ	07:05	0	O	1	0	33
CON 11	74	m	occ	08:00	0	A	0	NA	32
CON 12	89	m	occ	06:50	0	O	2	0	33
AD 1	67	f	occ	07:35	0	C	5	3	33
AD 2	82	m	par	03:35	0	C	6	3	44
AD 3	79	f	par	05:10	0	C	6	3	43
AD 4	85	f	occ	04:05	0	C	5	3	43
AD 5	77	m	par	06:35	0	C	6	3	44
AD 6	71	f	par	04:20	0	C	5	NA	33
AD 7	72	f	occ	06:30	0	C	4	3	43
AD 8	66	f	occ	04:12	0	C	6	3	33
AD 9	85	f	par	06:00	0	C	4	2	33
AD 10	67	m	par	08:20	0	C	5	2	33
AD 11	89	f	occ	04:00	0	C	6	3	43
AD 12	78	f	occ	08:15	0	C	5	3	33
AD 13	91	f	par	07:50	0	C	5	3	43

CAA, Aβ, Tau and CERAD staging according to Thal et al. [58], Braak & Braak [7, 8] and Mirra et al. [41]. ^$^Hereditary CAA case: β-amyloid precursor protein (APP) mutation E693Q causing hereditary cerebral hemorrhage with amyloidosis-Dutch type (HCHWA-D). NA: not available, PMD: postmortem delay

### The brain microvasculature of CAA type 1 displays a distinct proteomic profile with enrichment of multiple secreted proteins

**Fig. 2:**
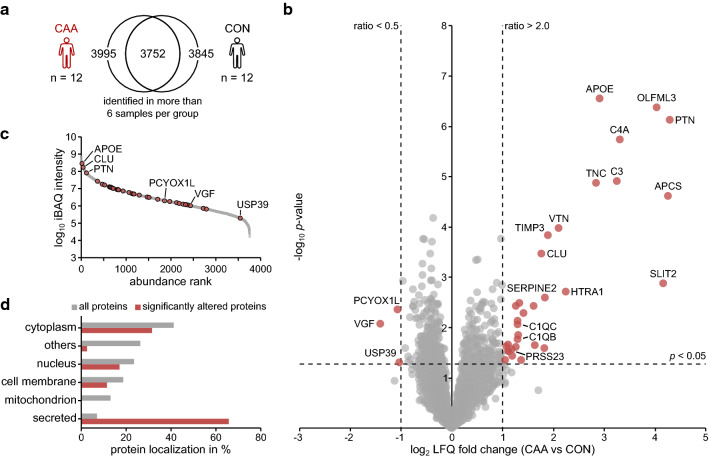
The brain microvascular proteome of patients with cerebral amyloid angiopathy. **a** Venn diagram demonstrating the overlap of 3752 proteins identified in at least 6 of the 12 samples from patients with cerebral amyloid angiopathy type 1 (CAA) and 6 of the 12 samples from control (CON) subjects. **b** Volcano plot illustrating log_2_ LFQ ratios (CAA vs CON) and -log10 *p* values of all quantified proteins. Red symbols (n = 35) indicate proteins with a significant change in abundance (*p* < 0.05, log_2_ ratio <  − 1.0/ > 1.0). **c** Abundance distribution according to the mean iBAQ intensity for each protein. Significantly altered proteins are indicated in red. **d** Protein localization of significantly altered proteins and of all identified proteins according to UniProt subcellular localization information

For the proteomic analysis, individual vessel preparations were subjected to LC/MS–MS followed by label-free quantification (LFQ) of protein abundances. As a first step, we performed a comparison of CAA samples with neurologically healthy controls. A total of 3752 proteins consistently identified by at least two unique peptides in at least six samples of each group were used for analysis (Fig. 2a and Additional file 2: Table 2). We found 35 proteins to be significantly altered with an abundance ratio of > two-fold (log_2_ ratio > 1) or < 0.5-fold (log_2_ ratio < − 1). The majority of them (n = 32) were enriched, 10 of which showed a ratio of > four-fold (Fig. 2b) revealing a strong overall tendency towards protein accumulation. As inferred from their mean intensity-based absolute quantification (iBAQ) intensities, significantly altered proteins were distributed across the full abundancy range (Fig. 2c). However, most of the proteins that were strongly enriched in CAA demonstrated high abundance, including apolipoprotein E (APOE), clusterin (CLU) and pleiotrophin (PTN). In contrast, the three depleted proteins prenylcysteine oxidase 1-like (PCYOX1L), VGF nerve growth factor inducible protein (VGF) and ubiquitin specific peptidase 39 (USP39) showed an overall lower abundance. A subcellular localization analysis based on UniProt database information revealed strong overrepresentation of secreted proteins (23 out of 35 proteins, 66%) (Fig. 2d). While some of these proteins such as APOE, CLU, serum amyloid P component (SAP, encoded by the APCS gene), vitronectin (VTN) and tissue inhibitor of metalloproteinases 3 (TIMP3) have previously been reported to be enriched in vascular amyloid deposits [18, 25, 38, 62], we also identified several novel proteins including serpine E2 (SERPINE2), olfactomedin-like protein 3 (OLFML3), the complement components C3, C1QB and C1QC, the extracellular matrix protein tenascin-C (TNC), the secreted glycoprotein slit guidance ligand 2 (SLIT2), PTN and the serine protease 23 (PRSS23) that have so far not been associated with CAA**.**

**Fig. 3:**
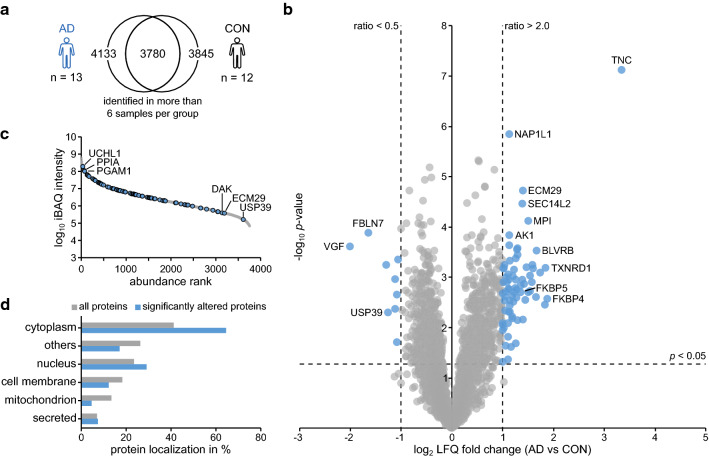
The brain microvascular proteome of patients with Alzheimer’s disease. **a** Venn diagram demonstrating the overlap of 3780 proteins identified in at least 6 of the 13 samples from patients with Alzheimer’s disease (AD) and 6 of the 12 samples from control (CON) subjects. **b** Volcano plot illustrating log_2_ LFQ ratios (AD vs CON) and − log10 *p* values of all quantified proteins. Blue symbols (n = 82) indicate proteins with a significant change in abundance (*p* < 0.05, log_2_ ratio <  − 1.0/ > 1.0). **c** Abundance distribution according to the mean iBAQ intensity for each protein. Significantly altered proteins are indicated in blue. The three most and least abundant proteins are labeled with gene names. **d** Protein localization of significantly altered proteins and of all identified proteins according to UniProt subcellular localization information

Next, we compared the proteome of brain microvessels from AD cases versus controls. A total of 3780 consistently identified proteins were used for analysis (Fig. 3a). Overall, we identified 82 proteins with a significantly altered abundance ratio of > two-fold (log_2_ ratio > 1) or < 0.5-fold (log_2_ ratio < − 1) (Fig. 3b and Additional file 3: Table 3). As in the CAA profile, significantly altered proteins distributed across a broad abundancy range (Fig. 3c). However, the overall extent of protein accumulation was substantially lower, with TNC as the single protein exceeding an abundance ratio of 4. Also, the subcellular localization profile of the 82 significantly altered proteins differed substantially from the CAA type 1 profile, in that there was an overrepresentation of cytoplasmic proteins but no enrichment of secreted proteins (Fig. 3d). This is in accord with the absence of detectable vascular Aβ deposits in the AD samples and indicates a less pronounced disruption of protein homeostasis.

**Fig. 4:**
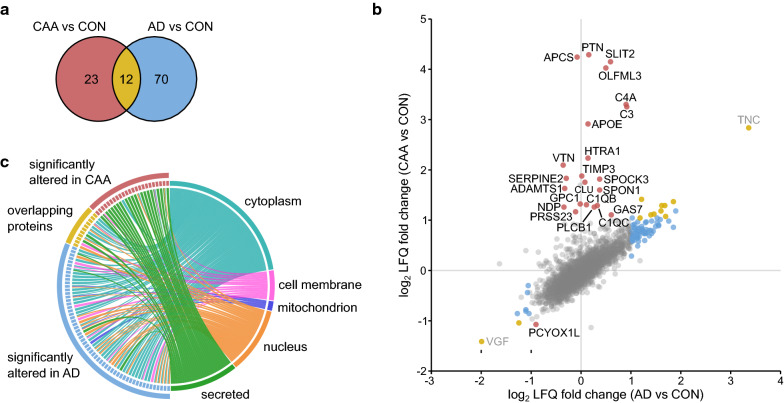
The CAA brain microvascular proteome displays a distinct profile independent of AD. **a** Venn diagram demonstrating the overlap between significantly altered proteins in CAA and AD (n = 12; *p* = 3.13 × 10^–12^). The overlapping proteins are displayed in yellow, the 23 and 70 proteins specifically altered in CAA and AD are shown in red and blue respectively. **b** Scatter plot of the log_2_ LFQ ratios in CAA versus CON against AD versus CON. Proteins exclusively altered in CAA are labeled with gene names, as are TNC and VGF. Significantly altered proteins are highlighted according to the colors in a, non-significantly altered proteins are shown in grey. **c** Circos diagram illustrating the subcellular localization information (UniProt) of the overlapping proteins and of the proteins specifically altered in CAA or AD

As a third step, we compared the microvascular proteomic profiles of CAA and AD considering proteins that surpassed the significance and fold change threshold (*p* < 0.05 and log_2_ fold change < − 1.0/ > 1.0) in either dataset. There was a significant overlap of 12 proteins, which further showed directional consistency, whereas 93 proteins did not overlap. Out of those, 23 and 70 proteins were specifically altered in CAA and AD, respectively (Fig. 4a). The comparison of individual LFQ ratios for CAA type 1 and AD (each compared to the control group) demonstrated a CAA type 1-specific cluster with high abundant changes whereas this was not observed for proteins constituting the AD-specific profile or for proteins that overlapped between the CAA type 1 and AD profiles (Fig. 4b). Subcellular localization analysis highlighted that the vast majority of proteins constituting the CAA-specific profile were annotated as secreted (21 out of 23 proteins, 91%) whereas proteins constituting the AD-specific profile or overlapping between the two profiles were distributed among all main subcellular localizations (Fig. 4c). Collectively, these findings define a distinct proteomic profile with enrichment of multiple secreted proteins in CAA type 1.

### CAA type 1 and CADASIL microvessels show a similar profile of protein accumulation

**Fig. 5:**
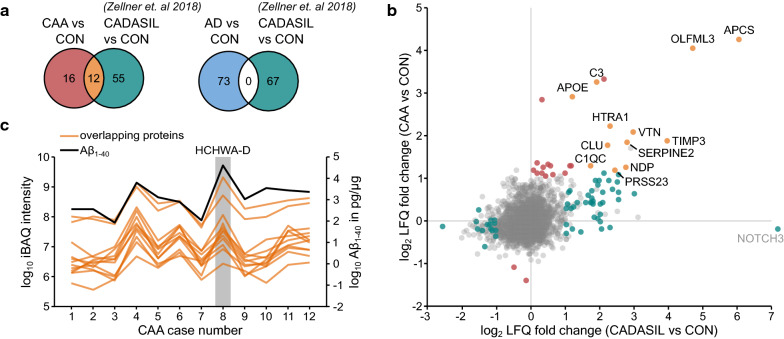
The CAA proteomic profile overlaps with the CADASIL proteomic profile. **a** Venn diagrams illustrating the overlap between proteins significantly altered in CAA (red) or AD patients (blue) with proteins significantly altered in CADASIL (teal) as reported in our earlier study [72]. A comparison between the CAA and CADASIL proteomic profiles results in an overlap of 12 proteins (*p* = 1.47 × 10^–13^) (orange), whereas no overlap was observed between the AD and CADASIL proteomic profiles. For better comparability, the CADASIL dataset was analyzed using the same significance and fold change thresholds as applied to the CAA and AD samples (*p* < 0.05 and log_2_ fold change <  − 1.0/ > 1.0). **b** Scatter plot of the log_2_ LFQ ratios in CAA versus CON against CADASIL versus CON. Significantly altered proteins are highlighted according to the colors in a, non-significantly altered proteins are shown in grey. Overlapping proteins are labeled with gene names, as is NOTCH3. The serine protease HTRA1 is highlighted in green. **c** Profile plot of Aβ_1-40_ levels (determined by ELISA) (black) and iBAQ intensities for the overlapping proteins (orange) in each individual CAA patient. For formal assessment of statistical correlations see Additional file 1: Figure 1. Case 8 represents a patient with hereditary cerebral hemorrhage with amyloidosis-Dutch type (HCHWA-D)

The accumulation of secreted proteins in the CAA type 1 profile was reminiscent of our recently determined brain microvascular proteome of CADASIL [72]. We, therefore, investigated a possible relationship by comparing proteins quantified in both datasets and exhibiting significantly altered abundance ratios (CAA type 1: n = 38, CADASIL: n = 67). This comparison revealed a significant overlap of 12 proteins (*p* = 1.47 × 10^–13^) representing almost one third of the CAA type 1 profile (Fig. 5a). Notably, there was no overlap between the AD and CADASIL profiles. The abundance changes of the proteins overlapping between CAA type 1 and CADASIL showed complete (100%) consistency in directionality, with all proteins enriched in both datasets (Fig. 5b). Moreover, there was a significant correlation (R = 0.58, *p* < 0.05) of the log_2_ LFQ ratios of the 12 overlapping proteins. Furthermore, all the overlapping proteins belonged to the secretory category providing additional evidence for shared mechanisms between CAA type 1 and CADASIL possibly related to the presence of abnormally folded proteins (Aβ and Notch3^ECD^, respectively) that characterize these conditions. Plotting the Aβ_1-40_ levels (as determined by ELISA) against the iBAQ intensities of the 12 proteins shared by the CAA and CADASIL profiles revealed significant correlations for all but 2 proteins (Fig. 5c and Additional file 4: Figure 1) indicating that their accumulation is indeed Aβ-dependent and not a general feature of disrupted vessel architecture. Of note, this included data from a patient with HCHWA-D, which were fully consistent with the results in sporadic CAA type 1 patients.

### Accumulation of HTRA1 and its substrates in CAA type 1

**Fig. 6:**
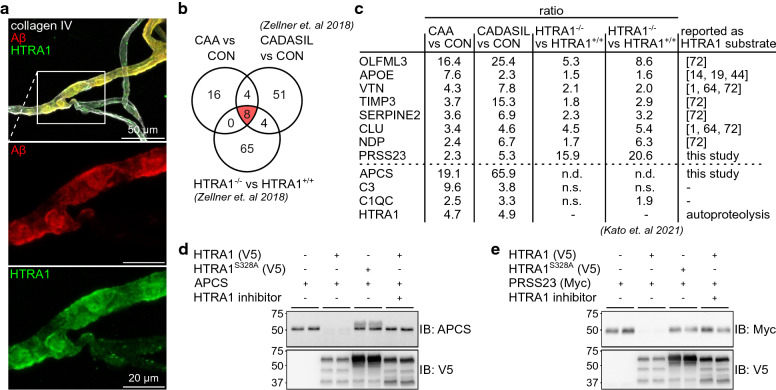
Accumulation of HTRA1 and its substrates in the CAA proteomic profile. **a** Immunofluorescence staining of an isolated capillary from a CAA patient (visualized by collagen IV staining) demonstrates a near complete co-localization of Aβ and HTRA1 immunoreactivity. **b** Venn diagram illustrating the overlap between proteins significantly altered in CAA with proteins significantly altered in CADASIL and in HTRA1 deficient mice as reported in our earlier study [72]. Eight of the shared proteins in CAA and CADASIL were also enriched in HTRA1 deficient mice (red filling). **c** Table with LFQ ratio information in the CAA, CADASIL and HTRA1 deficient proteomic profiles for the overlapping proteins**,** as for APCS, C3, C1QC and HTRA1. The latter four proteins are shared between CAA and CADASIL but not part of the HTRA1 deficient profile as they were not identified (APCS), not significantly altered (C3 and C1QC) or exclusively detected under HTRA1 wild-type conditions (HTRA1). Similar proteomic changes in HTRA1 deficient mice were recently reported by Kato et. al [34]. For most proteins in vitro cleavage data have been reported earlier. **d**, **e** APCS and PRSS23 are HTRA1 substrates. Shown are immunoblots of conditioned supernatants from HEK293 cells expressing PRSS23 (**d**) (detected via Myc-tag) or APCS (**e**), co-incubated with supernatants containing wild-type or active-site mutant (S328A) HTRA1 (detected via V5-tag) in the presence or absence of a HTRA1-specific inhibitor (5 μM). Molecular weight marker bands in kDa are indicated. The band above APCS in the S328A condition represent an unspecific cross-reactivity of the antibody. HTRA1 bands of lower molecular weight represent autoproteolysis fragments

Among the proteins shared by the CAA type 1 and CADASIL profiles was HTRA1, a serine protease genetically linked to hereditary SVD [23, 63] and further found to co-localize with Notch3 deposits in the vasculature of CADASIL patients [72]. Hence, we performed immunofluorescence co-staining of HTRA1 and Aβ in isolated capillaries from a CAA type 1 sample, which revealed a near-complete co-localization (Fig. 6a). This confirms and extends previous findings from Hondius et al. [25] and suggested a recruitment of HTRA1 to pathological Aβ deposits including a possible sequestration process. To investigate whether the overlap between CAA type 1 and CADASIL could be attributed to HTRA1 substrate enrichment and thus indicate a loss-of-function mechanism as proposed for CADASIL [72], we conducted a three-way comparison between the CAA type 1, the CADASIL and our published HTRA1^−/−^ mouse microvessel profile. Notably, proteins accumulating in HTRA1^−/−^ mice, can be considered putative HTRA1 substrates, as these mice do not develop protein deposits. Eight of the 12 proteins that were enriched in both the CAA type 1 and CADASIL profiles (OLFML3, APOE, VTN, TIMP3, SERPINE2, CLU, NDP and PRSS23) also showed increased abundancy in HTRA1^−/−^ microvessels (Fig. 6b, c) suggesting that these proteins indeed represent HTRA1 substrates. HTRA1 was exclusively found in the wild-type animals and thus did not contribute to the signature. The same applies for APCS, which was not identified in mice. Thus, only C3 and C1QC were consistently quantified but showed no significant changes in HTRA1^−/−^ mice. Importantly, this finding was fully confirmed in a recent independent study on HTRA1-deficient mice [34]. Also, seven of the eight proteins (OLFML3, APOE, VTN, TIMP3, SERPINE2, CLU and NDP) have previously been shown in proteolysis assays to be HTRA1 substrates [1, 9, 14, 44, 64, 72] (Fig. 6c). To explore the candidacy of APCS and PRSS23 as potential substrates of HTRA1, we next performed cell-based cleavage assays: APCS was the most strongly enriched protein in the overlap signature; PRSS23 shares features with HTRA1 in that it represents a member of the family of chymotrypsin-type serine proteases and is primarily secreted by astrocytes [53, 61]. We expressed epitope-tagged constructs in HEK293 cells, a commonly used approach for analyzing HTRA1 proteolytic activity in a cellular setting [4, 72], co-incubated substrate- and protease-containing supernatants and investigated the resulting protein levels using Western Blotting (Fig. 6d, e). While HTRA1 was secreted as a ~ 55-kDa protein (bands of lower molecular weight represent autoproteolysis products), APCS was detected as a single band of ~ 51 kDa (26-kDa full-length protein plus the 25-kDa epitope tag) and PRSS23 migrated as a 37-kDa band (most likely generated from the 45-kDa full-length protein by intracellular cleavage at a furin consensus site). Upon co-incubation with wild-type HTRA1, APCS and PRSS23 levels were strongly reduced close to detection limit, whereas this was not the case upon co-incubation with HTRA1^S328A^, an artificial mutation which eliminates the catalytic serine residue resulting in a complete loss of proteolytic activity, or in the presence of a HTRA1-specific inhibitor. We thus identified APCS and PRSS23 as novel HTRA1 substrates providing further support for a role of functional deficiency of HTRA1 in CAA type 1 pathogenesis.

## Discussion

Applying untargeted proteomics to isolated parenchymal microvessels from post-mortem CAA type 1 brain samples, we identified a distinct profile that was characterized by an enrichment of multiple secreted proteins and substantially overlapped with the proteomic profile of CADASIL, a genetic form of cerebral SVD. This overlap, which was not observed in a comparison with the brain microvascular profile of AD patients lacking vascular amyloid pathology, can for the most part be attributed to the accumulation of HTRA1 and several of its substrates. HTRA1 is a protease implicated in the regulation of extracellular protein homeostasis and centrally involved in the pathogenesis of yet another form of hereditary cerebral SVD [23, 63]. Collectively, these findings indicate a critical role of impaired HTRA1-mediated protein degradation in the CAA type 1 microvasculature and suggest shared mechanisms across different types of cerebral SVD.

In many respects, the current study represents a major advancement over previous proteomic investigations on CAA [18, 25, 28, 38]. Aside from focusing on isolated brain microvessels we determined the proteome of CAA patients with pronounced capillary Aβ pathology. Generating proteomic data on a large sample of patients and controls in unprecedented depth and using label-free quantification, we identified a distinct set of proteins with strongly increased abundance. This profile recapitulates the enrichment of proteins reported in earlier studies (APCS, APOE, CLU, HTRA1, NDP, TIMP3, VTN), while also revealing an accumulation of several proteins so far not associated with CAA including PTN, SLIT2, TNC, the complement components C3, C1QB and C1QC as well as OLFML3, PRSS23 and SERPINE2. For a considerable fraction of these proteins we provide evidence for a link to HTRA1 proteolytic function (see below), but other proteins including PTN, SLIT2, TNC and the complement components might independently contribute to pathogenesis via alternative mechanisms: PTN, a pericyte-secreted neurotrophic growth factor that is exclusively expressed in the brain, has been shown to protect neurons against ischemic and excitotoxic injury [46]. Its recruitment to microvascular Aβ deposits might result in functional inactivation of PTN and thus enhance the adverse effects of vascular dysfunction on neuronal integrity in CAA. The extracellular signaling factor SLIT2, has been implicated in angiogenesis and the regulation of vascular permeability [30, 35] and might thus contribute to the loss of BBB integrity in CAA [36]. TNC, an ECM glycoprotein linked to inflammatory processes in the brain, has been shown to associate with cored Aβ plaques as well as vascular Aβ deposits in AD patients and cognitively normal elderly individuals showing plaque pathology [40] and might represent an immune response modulator during CAA pathogenesis [70]. Support for a role of inflammatory processes further comes from the increased abundance of C3, C4A, C1QB and C1QC, components of the classical and alternative complement pathways.

We found the proteomic profile of CAA type 1 to show remarkable overlap with the profile of brain microvessels in CADASIL, another SVD caused by protein misfolding. Like CAA, CADASIL is characterized by vascular protein deposits, whose primary constituent, however, is Notch3^ECD^, a receptor fragment generated at the surface of mural cells. Notch3^ECD^ deposits differ from Aβ deposits regarding structure [72] and ultrastructure [31]. As such, the enrichment of a shared set of proteins, most notably HTRA1, is noteworthy. HTRA1 is implicated in ATP-independent protein quality control across multiple species including removal of misfolded or mislocalized polypeptides [15]. The extensive co-localization of HTRA1 with Aβ deposits in isolated brain capillaries is in accord with this concept, although the mechanisms of HTRA1 recruitment remain to be identified. HTRA1 accumulation related to vascular Aβ pathology has previously been reported on a qualitative level in human brain vasculature obtained by microdissection [25] and in vessel-enriched fractions from Tg-SwDI mice [52] and TgDI rats [51]. Indeed, a recent review of available literature proposed there is an overlap between the profiles of CAA and CADASIL, which, however, was not formally assessed [71].

The majority of highly enriched proteins shared by our CAA type 1 and CADASIL profiles also accumulate in brain microvessels of Htra1 knockout mice [34, 72] and thus represent putative HTRA1 substrates. Accordingly, HTRA1-mediated processing of a number of these proteins has been demonstrated by us and others in *in-vitro* cleavage assays [1, 9, 14, 44, 64, 72]. We now expand on this list by demonstrating HTRA1-dependent processing of APCS and PRSS23 providing further support for an important role of HTRA1 activity in SVD pathogenesis. Several observations suggest that the accumulating proteins we found here to overlap between the CAA type 1 and CADASIL profiles and to possibly represent a HTRA1 loss-of-function signature contribute to the pathophysiology of CAA type 1 and are not a mere reflection of vessel degeneration. First, inactivating mutations in HTRA1 have previously been shown to cause degenerative changes in brain microvessels [23, 47, 54] thus placing loss of HTRA1 function upstream of vessel damage. Second, we did not find these proteins to accumulate in brain microvessels from patients with sporadic SVD, which likewise show degenerative changes (unpublished results). Third, some of the HTRA1 substrates including TIMP3 have previously been shown to contribute to SVD pathogenesis [10, 11]. Still, the precise mechanisms linking HTRA1 to vascular degeneration in CAA type 1 remain unknown. Notably, a recent large-scale sequencing study in the general population has uncovered an association of rare loss-of-function HTRA1 variants with white matter lesion volume, a quantitative marker of cerebral SVD, indicating a potential role of HTRA1 also in sporadic SVD forms [37].

In light of our findings, we propose a refined model for the molecular function of HTRA1. We hypothesize that, under physiological conditions, HTRA1 degrades excess proteins thus safeguarding extracellular proteostasis. Support for this comes from studies demonstrating HTRA1-mediated degradation of oligomeric/fibrillar Aβ and tau species [19, 49, 56] and from previous findings showing an accumulation of multiple constituents of the vascular ECM in Htra1-deficient mice [34, 72]. In CAA type 1 and CADASIL, HTRA1 is recruited into the vascular Aβ and Notch3^ECD^ deposits characterizing these vasculopathies. We propose, this occurs to remove misfolded and excess polypeptides. Progressive growth of the deposits may result in a sequestration and depletion of HTRA1 from the extracellular environment leading to functional inactivation, substrate accumulation and eventually disruption of proteostasis [22]. While direct evidence for this concept remains to be presented, we would argue that the accumulation of multiple HTRA1 substrates supports our hypothesis. Upon first sight, HTRA1-related genetic cerebral SVDs lacking distinct protein deposits seem to be at odds with this concept. Of note, however, brain vessels of these patients exhibit prominent abnormalities in angioarchitecture including intimal thickening and ECM expansion [23, 47] consistent with altered proteostasis. An alternative explanation for the vascular structural alterations would be dysregulated TGFβ signaling [23, 29, 39]. However, we found constituents of the TGFβ pathway to be either not detected in a sufficient number of samples or abundance changes did not reach significance. A proteomic investigation of vessels from patients with HTRA1-related familial cerebral SVD was not feasible due to a lack of appropriate autopsy material.

While HTRA1 is present in human senile plaques [19], it was not enriched in brain microvessels from our AD patients. Accordingly, there was no accumulation of HTRA1 substrates that we found here to be shared between the CAA type 1 and CADASIL profile. Still, we found the CAA type 1 and AD profiles to show some overlap, evidencing shared molecular pathways independent of vascular Aβ deposition. Overall, this overlap was less pronounced in terms of the abundance ratios of individual proteins, which further belong to various cellular pathways. As such, the underlying mechanisms may be more complex.

Specific strengths of this study include the application of untargeted and quantitative proteomics to isolated microvessel preparations from a large sample of well characterized cases of CAA type 1 patients, healthy controls, and AD patients without vascular pathology, and cross-referencing of proteomics results to those from CADASIL patients. However, this study also has limitations. In particular, the transferability of our findings to CAA type 2 patients is unclear and the mechanistic details of the recruitment of HTRA1 to pathological deposits and its functional consequences remain to be determined. Still, our findings argue for a critical role of HTRA1 in CAA type 1-affected microvessels and reveal an unanticipated molecular link between CAA type 1 and other forms of microvascular disease thus emphasizing the importance of this pathway in brain health.

## Supplementary Information

**Additional file 1**. Levels of the amyloid species Aβ_1-40_ and Aβ_1-42_ in the microvessel extracts determined by ELISA.

**Additional file 2**. Results of the proteomic analysis of CAA brain microvessel extracts.

**Additional file 3**. Results of the proteomic analysis of AD brain microvessel extracts.

**Additional file 4**. Shared proteins between CAA and CADASIL reveal high correlation with Aβ_1-40_. Scatter plots of log_10_ iBAQ intensities and log_10_ Aβ_1-40_ intensities (determined by ELISA) for the 12 proteins enriched in CAA and CADASIL microvessel extracts.* p*-values and correlation coefficients were calculated via linear regression analysis.

## Data Availability

The mass spectrometry proteomics data have been deposited to the ProteomeXchange Consortium via the PRIDE partner repository with the dataset identifers PXD029380.

## References

[1] An E, Sen S, Park SK, Gordish-Dressman H, Hathout Y (2010). Identification of novel substrates for the serine protease HTRA1 in the human RPE secretome. Invest Ophthalmol Vis Sci.

[2] Attems J, Jellinger K, Thal DR, Van Nostrand W (2011). Review: sporadic cerebral amyloid angiopathy. Neuropathol Appl Neurobiol.

[3] Attems J, Jellinger KA (2004). Only cerebral capillary amyloid angiopathy correlates with Alzheimer pathology—a pilot study. Acta Neuropathol.

[4] Beaufort N, Scharrer E, Kremmer E, Lux V, Ehrmann M, Huber R, Houlden H, Werring D, Haffner C, Dichgans M (2014). Cerebral small vessel disease-related protease HtrA1 processes latent TGF-beta binding protein 1 and facilitates TGF-beta signaling. Proc Natl Acad Sci USA.

[5] Bian Y, Zheng R, Bayer FP, Wong C, Chang YC, Meng C, Zolg DP, Reinecke M, Zecha J, Wiechmann S (2020). Robust, reproducible and quantitative analysis of thousands of proteomes by micro-flow LC-MS/MS. Nat Commun.

[6] Bourassa P, Tremblay C, Schneider JA, Bennett DA, Calon F (2019). Beta-amyloid pathology in human brain microvessel extracts from the parietal cortex: relation with cerebral amyloid angiopathy and Alzheimer's disease. Acta Neuropathol.

[7] Braak H, Braak E (1991). Neuropathological stageing of Alzheimer-related changes. Acta Neuropathol.

[8] Braak H, Braak E (1995). Staging of Alzheimer's disease-related neurofibrillary changes. Neurobiol Aging.

[9] Cabrera AC, Melo E, Roth D, Topp A, Delobel F, Stucki C, Chen CY, Jakob P, Banfai B, Dunkley T (2017). HtrA1 activation is driven by an allosteric mechanism of inter-monomer communication. Sci Rep.

[10] Capone C, Cognat E, Ghezali L, Baron-Menguy C, Aubin D, Mesnard L, Stohr H, Domenga-Denier V, Nelson MT, Joutel A (2016). Reducing Timp3 or vitronectin ameliorates disease manifestations in CADASIL mice. Ann Neurol.

[11] Capone C, Dabertrand F, Baron-Menguy C, Chalaris A, Ghezali L, Domenga-Denier V, Schmidt S, Huneau C, Rose-John S, Nelson MT (2016). Mechanistic insights into a TIMP3-sensitive pathway constitutively engaged in the regulation of cerebral hemodynamics. Elife.

[12] Carare RO, Hawkes CA, Jeffrey M, Kalaria RN, Weller RO (2013). Review: cerebral amyloid angiopathy, prion angiopathy, CADASIL and the spectrum of protein elimination failure angiopathies (PEFA) in neurodegenerative disease with a focus on therapy. Neuropathol Appl Neurobiol.

[13] Charidimou A, Boulouis G, Gurol ME, Ayata C, Bacskai BJ, Frosch MP, Viswanathan A, Greenberg SM (2017). Emerging concepts in sporadic cerebral amyloid angiopathy. Brain.

[14] Chu Q, Diedrich JK, Vaughan JM, Donaldson CJ, Nunn MF, Lee KF, Saghatelian A (2016). HtrA1 proteolysis of ApoE In vitro is allele selective. J Am Chem Soc.

[15] Clausen T, Kaiser M, Huber R, Ehrmann M (2011). HTRA proteases: regulated proteolysis in protein quality control. Nat Rev Mol Cell Biol.

[16] Cox J, Hein MY, Luber CA, Paron I, Nagaraj N, Mann M (2014). Accurate proteome-wide label-free quantification by delayed normalization and maximal peptide ratio extraction, termed MaxLFQ. Mol Cell Proteom.

[17] Dichgans M, Pulit SL, Rosand J (2019). Stroke genetics: discovery, biology, and clinical applications. Lancet Neurol.

[18] Endo Y, Hasegawa K, Nomura R, Arishima H, Kikuta KI, Yamashita T, Inoue Y, Ueda M, Ando Y, Wilson MR (2019). Apolipoprotein E and clusterin inhibit the early phase of amyloid-beta aggregation in an in vitro model of cerebral amyloid angiopathy. Acta Neuropathol Commun.

[19] Grau S, Baldi A, Bussani R, Tian X, Stefanescu R, Przybylski M, Richards P, Jones SA, Shridhar V, Clausen T (2005). Implications of the serine protease HtrA1 in amyloid precursor protein processing. Proc Natl Acad Sci USA.

[20] Greenberg SM, Bacskai BJ, Hernandez-Guillamon M, Pruzin J, Sperling R, van Veluw SJ (2020). Cerebral amyloid angiopathy and Alzheimer disease—one peptide, two pathways. Nat Rev Neurol.

[21] Grinberg LT, Thal DR (2010). Vascular pathology in the aged human brain. Acta Neuropathol.

[22] Haffner C, Malik R, Dichgans M (2016). Genetic factors in cerebral small vessel disease and their impact on stroke and dementia. J Cereb Blood Flow Metab.

[23] Hara K, Shiga A, Fukutake T, Nozaki H, Miyashita A, Yokoseki A, Kawata H, Koyama A, Arima K, Takahashi T (2009). Association of HTRA1 mutations and familial ischemic cerebral small-vessel disease. N Engl J Med.

[24] Hecht M, Kramer LM, von Arnim CAF, Otto M, Thal DR (2018). Capillary cerebral amyloid angiopathy in Alzheimer's disease: association with allocortical/hippocampal microinfarcts and cognitive decline. Acta Neuropathol.

[25] Hondius DC, Eigenhuis KN, Morrema THJ, van der Schors RC, van Nierop P, Bugiani M, Li KW, Hoozemans JJM, Smit AB, Rozemuller AJM (2018). Proteomics analysis identifies new markers associated with capillary cerebral amyloid angiopathy in Alzheimer's disease. Acta Neuropathol Commun.

[26] Hughes CS, Sorensen PH, Morin GB (2019). A standardized and reproducible proteomics protocol for bottom-up quantitative analysis of protein samples using SP3 and mass spectrometry. Methods Mol Biol.

[27] Iadecola C, Duering M, Hachinski V, Joutel A, Pendlebury ST, Schneider JA, Dichgans M (2019). Vascular cognitive impairment and dementia: JACC scientific expert panel. J Am Coll Cardiol.

[28] Inoue Y, Ueda M, Tasaki M, Takeshima A, Nagatoshi A, Masuda T, Misumi Y, Kosaka T, Nomura T, Mizukami M (2017). Sushi repeat-containing protein 1: a novel disease-associated molecule in cerebral amyloid angiopathy. Acta Neuropathol.

[29] Ito S, Takao M, Fukutake T, Hatsuta H, Funabe S, Ito N, Shimoe Y, Niki T, Nakano I, Fukayama M (2016). Histopathologic analysis of cerebral autosomal recessive arteriopathy with subcortical infarcts and leukoencephalopathy (CARASIL): a report of a new genetically confirmed case and comparison to 2 previous cases. J Neuropathol Exp Neurol.

[30] Jones CA, Nishiya N, London NR, Zhu W, Sorensen LK, Chan AC, Lim CJ, Chen H, Zhang Q, Schultz PG (2009). Slit2-Robo4 signalling promotes vascular stability by blocking Arf6 activity. Nat Cell Biol.

[31] Joutel A, Andreux F, Gaulis S, Domenga V, Cecillon M, Battail N, Piga N, Chapon F, Godfrain C, Tournier-Lasserve E (2000). The ectodomain of the Notch3 receptor accumulates within the cerebrovasculature of CADASIL patients. J Clin Invest.

[32] Kalaria RN (2016). Neuropathological diagnosis of vascular cognitive impairment and vascular dementia with implications for Alzheimer's disease. Acta Neuropathol.

[33] Kamp JA, Moursel LG, Haan J, Terwindt GM, Lesnik Oberstein SA, van Duinen SG, van Roon-Mom WM (2014). Amyloid beta in hereditary cerebral hemorrhage with amyloidosis-Dutch type. Rev Neurosci.

[34] Kato T, Manabe RI, Igarashi H, Kametani F, Hirokawa S, Sekine Y, Fujita N, Saito S, Kawashima Y, Hatano Y (2021). Candesartan prevents arteriopathy progression in cerebral autosomal recessive arteriopathy with subcortical infarcts and leukoencephalopathy model. J Clin Invest.

[35] Li JC, Han L, Wen YX, Yang YX, Li S, Li XS, Zhao CJ, Wang TY, Chen H, Liu Y (2015). Increased permeability of the blood-brain barrier and Alzheimer's disease-like alterations in slit-2 transgenic mice. J Alzheimers Dis.

[36] Magaki S, Tang Z, Tung S, Williams CK, Lo D, Yong WH, Khanlou N, Vinters HV (2018). The effects of cerebral amyloid angiopathy on integrity of the blood-brain barrier. Neurobiol Aging.

[37] Malik R, Beaufort N, Frerich S, Gesierich B, Georgakis MK, Rannikmae K, Ferguson AC, Haffner C, Traylor M, Ehrmann M (2021). Whole-exome sequencing reveals a role of HTRA1 and EGFL8 in brain white matter hyperintensities. Brain.

[38] Manousopoulou A, Gatherer M, Smith C, Nicoll JAR, Woelk CH, Johnson M, Kalaria R, Attems J, Garbis SD, Carare RO (2017). Systems proteomic analysis reveals that clusterin and tissue inhibitor of metalloproteinases 3 increase in leptomeningeal arteries affected by cerebral amyloid angiopathy. Neuropathol Appl Neurobiol.

[39] Masliah E, Ho G, Wyss-Coray T (2001). Functional role of TGF beta in Alzheimer's disease microvascular injury: lessons from transgenic mice. Neurochem Int.

[40] Mi Z, Halfter W, Abrahamson EE, Klunk WE, Mathis CA, Mufson EJ, Ikonomovic MD (2016). Tenascin-C is associated with cored amyloid-beta plaques in alzheimer disease and pathology burdened cognitively normal elderly. J Neuropathol Exp Neurol.

[41] Mirra SS, Heyman A, McKeel D, Sumi SM, Crain BJ, Brownlee LM, Vogel FS, Hughes JP, van Belle G, Berg L (1991). The consortium to establish a registry for Alzheimer's disease (CERAD). Part II. Standardization of the neuropathologic assessment of Alzheimer's disease. Neurology.

[42] Monet-Lepretre M, Haddad I, Baron-Menguy C, Fouillot-Panchal M, Riani M, Domenga-Denier V, Dussaule C, Cognat E, Vinh J, Joutel A (2013). Abnormal recruitment of extracellular matrix proteins by excess Notch3 ECD: a new pathomechanism in CADASIL. Brain.

[43] Morris AW, Carare RO, Schreiber S, Hawkes CA (2014). The Cerebrovascular basement membrane: role in the clearance of beta-amyloid and cerebral amyloid angiopathy. Front Aging Neurosci.

[44] Munoz SS, Li H, Ruberu K, Chu Q, Saghatelian A, Ooi L, Garner B (2018). The serine protease HtrA1 contributes to the formation of an extracellular 25-kDa apolipoprotein E fragment that stimulates neuritogenesis. J Biol Chem.

[45] Nelson AR, Sweeney MD, Sagare AP, Zlokovic BV (2016). Neurovascular dysfunction and neurodegeneration in dementia and Alzheimer's disease. Biochim Biophys Acta.

[46] Nikolakopoulou AM, Montagne A, Kisler K, Dai Z, Wang Y, Huuskonen MT, Sagare AP, Lazic D, Sweeney MD, Kong P (2019). Pericyte loss leads to circulatory failure and pleiotrophin depletion causing neuron loss. Nat Neurosci.

[47] Oide T, Nakayama H, Yanagawa S, Ito N, Ikeda S, Arima K (2008). Extensive loss of arterial medial smooth muscle cells and mural extracellular matrix in cerebral autosomal recessive arteriopathy with subcortical infarcts and leukoencephalopathy (CARASIL). Neuropathology.

[48] Pantoni L (2010). Cerebral small vessel disease: from pathogenesis and clinical characteristics to therapeutic challenges. Lancet Neurol.

[49] Poepsel S, Sprengel A, Sacca B, Kaschani F, Kaiser M, Gatsogiannis C, Raunser S, Clausen T, Ehrmann M (2015). Determinants of amyloid fibril degradation by the PDZ protease HTRA1. Nat Chem Biol.

[50] Rasmussen MK, Mestre H, Nedergaard M (2018). The glymphatic pathway in neurological disorders. Lancet Neurol.

[51] Schrader JM, Xu F, Van Nostrand WE (2021). Distinct brain regional proteome changes in the rTg-DI rat model of cerebral amyloid angiopathy. J Neurochem.

[52] Searcy JL, Le Bihan T, Salvadores N, McCulloch J, Horsburgh K (2014). Impact of age on the cerebrovascular proteomes of wild-type and Tg-SwDI mice. PLoS ONE.

[53] Sharma K, Schmitt S, Bergner CG, Tyanova S, Kannaiyan N, Manrique-Hoyos N, Kongi K, Cantuti L, Hanisch UK, Philips MA (2015). Cell type- and brain region-resolved mouse brain proteome. Nat Neurosci.

[54] Shiga A, Nozaki H, Yokoseki A, Nihonmatsu M, Kawata H, Kato T, Koyama A, Arima K, Ikeda M, Katada S (2011). Cerebral small-vessel disease protein HTRA1 controls the amount of TGF-beta1 via cleavage of proTGF-beta1. Hum Mol Genet.

[55] Sun Y, Vandenbriele C, Kauskot A, Verhamme P, Hoylaerts MF, Wright GJ (2015). A human platelet receptor protein microarray identifies the high affinity immunoglobulin e receptor subunit alpha (FcepsilonR1alpha) as an Activating Platelet Endothelium Aggregation Receptor 1 (PEAR1) ligand. Mol Cell Proteom.

[56] Tennstaedt A, Popsel S, Truebestein L, Hauske P, Brockmann A, Schmidt N, Irle I, Sacca B, Niemeyer CM, Brandt R (2012). Human high temperature requirement serine protease A1 (HTRA1) degrades tau protein aggregates. J Biol Chem.

[57] Thal DR, Capetillo-Zarate E, Larionov S, Staufenbiel M, Zurbruegg S, Beckmann N (2009). Capillary cerebral amyloid angiopathy is associated with vessel occlusion and cerebral blood flow disturbances. Neurobiol Aging.

[58] Thal DR, Ghebremedhin E, Rub U, Yamaguchi H, Del Tredici K, Braak H (2002). Two types of sporadic cerebral amyloid angiopathy. J Neuropathol Exp Neurol.

[59] Thal DR, Grinberg LT, Attems J (2012). Vascular dementia: different forms of vessel disorders contribute to the development of dementia in the elderly brain. Exp Gerontol.

[60] Thal DR, Walter J, Saido TC, Fandrich M (2015). Neuropathology and biochemistry of Abeta and its aggregates in Alzheimer's disease. Acta Neuropathol.

[61] Tushaus J, Muller SA, Kataka ES, Zaucha J, Sebastian Monasor L, Su M, Guner G, Jocher G, Tahirovic S, Frishman D (2020). An optimized quantitative proteomics method establishes the cell type-resolved mouse brain secretome. EMBO J.

[62] Verbeek MM, Otte-Holler I, Veerhuis R, Ruiter DJ, De Waal RM (1998). Distribution of A beta-associated proteins in cerebrovascular amyloid of Alzheimer's disease. Acta Neuropathol.

[63] Verdura E, Herve D, Scharrer E, Amador Mdel M, Guyant-Marechal L, Philippi A, Corlobe A, Bergametti F, Gazal S, Prieto-Morin C (2015). Heterozygous HTRA1 mutations are associated with autosomal dominant cerebral small vessel disease. Brain.

[64] Vierkotten S, Muether PS, Fauser S (2011). Overexpression of HTRA1 leads to ultrastructural changes in the elastic layer of Bruch's membrane via cleavage of extracellular matrix components. PLoS ONE.

[65] Vinters HV, Zarow C, Borys E, Whitman JD, Tung S, Ellis WG, Zheng L, Chui HC (2018). Review: Vascular dementia: clinicopathologic and genetic considerations. Neuropathol Appl Neurobiol.

[66] Vonsattel JP, Myers RH, Hedley-Whyte ET, Ropper AH, Bird ED, Richardson EP (1991). Cerebral amyloid angiopathy without and with cerebral hemorrhages: a comparative histological study. Ann Neurol.

[67] Wang M, Zhao Y, Zhang B (2015). Efficient test and visualization of multi-set intersections. Sci Rep.

[68] Wardlaw JM, Smith C, Dichgans M (2019). Small vessel disease: mechanisms and clinical implications. Lancet Neurol.

[69] Weller RO, Hawkes CA, Kalaria RN, Werring DJ, Carare RO (2015). White matter changes in dementia: role of impaired drainage of interstitial fluid. Brain Pathol.

[70] Wiemann S, Reinhard J, Faissner A (2019). Immunomodulatory role of the extracellular matrix protein tenascin-C in neuroinflammation. Biochem Soc Trans.

[71] Young KZ, Xu G, Keep SG, Borjigin J, Wang MM (2020). Overlapping protein accumulation profiles of CADASIL and CAA: Is there a common mechanism driving cerebral small-vessel disease?. Am J Pathol.

[72] Zellner A, Scharrer E, Arzberger T, Oka C, Domenga-Denier V, Joutel A, Lichtenthaler SF, Muller SA, Dichgans M, Haffner C (2018). CADASIL brain vessels show a HTRA1 loss-of-function profile. Acta Neuropathol.

