# Supplementary material for: Proteomic profiling in cerebral amyloid angiopathy reveals an overlap with CADASIL highlighting accumulation of HTRA1 and its substrates
Source: Acta Neuropathol Commun. 2022 Jan 24;10:6. doi: 10.1186/s40478-021-01303-6 (PMC8785498; doi:10.1186/s40478-021-01303-6)
Supplement: Supplementary file 4 — Additional file 4. Shared proteins between CAA and CADASIL reveal high correlation with Aβ1-40. Scatter plots of log10 iBAQ intensities and log10 Aβ1-40 intensities (determined by ELISA) for the 12 proteins enriched in CAA and CADASIL microvessel extracts. p-values and correlation coefficients were calculated via linear regression analysis. [file 40478_2021_1303_MOESM4_ESM.pdf]

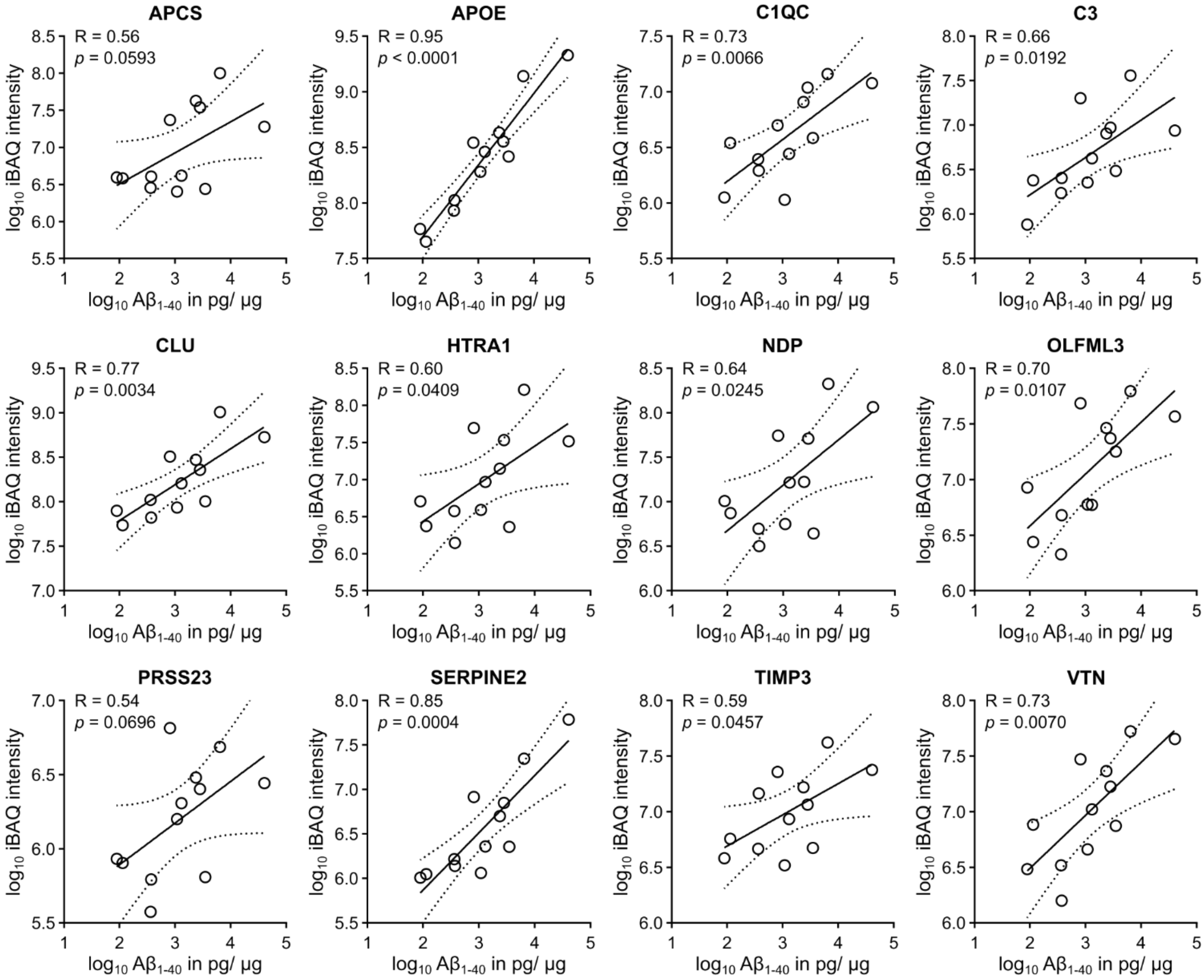

Fig. S1 Shared proteins between CAA and CADASIL reveal high correlation with A$\beta_{1\text{-}40}$

Scatter plots of $\log_{10}$ iBAQ intensities and $\log_{10}$ A$\beta_{1\text{-}40}$ intensities (determined by ELISA) for the 12 proteins enriched in CAA and CADASIL microvessel extracts. *p*-values and correlation coefficients were calculated via linear regression analysis.
